# THE ASSOCIATIONS OF LATE-LIFE BLOOD PRESSURE WITH CERAD AND BRAAK STAGES: FINDINGS FROM THE NACC DATASET

**DOI:** 10.1093/geroni/igae098.0730

**Published:** 2024-12-31

**Authors:** Mo-kyung Sin, N Maritza Dowling, Jeffrey Roseman, Ali Ahmed, Edward Zamrini

**Affiliations:** Seattle University, Seattle University, Washington, United States; The George Washington University, Washington, District of Columbia, United States; University of Alabama at Birmingham, Birmingham, Alabama, United States; Washington DC Veterans Medical Center, VA Medical Center, District of Columbia, United States; Irvine Clinical Research, Irvine, California, United States

## Abstract

CERAD and Braak staging are two major scoring systems for quantifying neuropathological features of Alzheimer’s disease (AD). Midlife hypertension is a risk factor for AD, but less is known about the relationships between late-life systolic blood pressure (SBP) with CERAD and Braak staging, the examination of which was the objective of the current study. This study analyzed data from 5,160 participants in the National Alzheimer’s Coordinating Center dataset which had data on 4 longitudinal SBP measurements before death and on CERAD (n=2,600) and Braak (n=2,560) autopsy data. CERAD score was dichotomized into moderate/severe versus none/sparse; Braak stage was dichotomized into groups: III-VI vs 0-II. Logistic regression models were used to examine odds of higher CERAD or Braak staging associated with SBP ≥130mmHg (mean of 4 late-life measures). In a model that controlled for age at death, last BP to death (in years), and sex, a late-life mean SBP ≥130mmHg compared to SBP < 130mmHg was associated with an estimated odds ratio for a more severe CERAD score of 1.13 (95% CI: 1.00, 1.26). In a model that also controlled for antihypertensive medications and APOE ε4 status, SBP ≥130mmHg was associated with a slightly increased estimated odd for a more severe CERAD score of 1.15 (95% CI: 1.02, 1.30). We found a direct association between late-life mean SBP with CERAD score, but not with Braak stage. This analysis supports the importance of maintaining control of BP into late life as a strategy to slow the rate of progression of AD pathology.

